# Effectiveness of Surgical Treatment in Carpal Tunnel Syndrome Mini-Incision Using MIS-CTS Kits: A Cadaveric Study

**DOI:** 10.1155/2020/8278054

**Published:** 2020-02-14

**Authors:** Wongthawat Liawrungrueang, Sunton Wongsiri

**Affiliations:** Department of Orthopaedic Surgery, Faculty of Medicine, Prince of Songkla University, Hat Yai, Thailand

## Abstract

*Introduction*. Carpal tunnel syndrome (CTS) is caused by the compression of the median nerves in the wrist. Patients have pain and numbness in the hands. According to the records of Songklanagarind Hospital from 2015 to 2018, of 800 patients, 196 or 24.5% were treated with surgery. The novel tool of minimally invasive surgery for carpal tunnel syndrome (MIS-CTS) was developed to improve effectiveness and safety.

**Purpose:**

This study was performed to the effectiveness of visualization during surgery and the complete release of the transverse carpal ligament (TCL) and also the safety of using the MIS-CTS kits.

**Methods:**

Twenty fresh cadaveric forearms had surgery. Surgical techniques were (1) incision 15–18 mm at palmar hand; (2) the scissors and the navigator were inserted to create working space underneath the palmar aponeurosis; (3) the visual enhancer was inserted. The visual enhancer improves the visual field by shielding the soft tissue around the operative field; (4) the TCL was cut at the distal TCL by surgery scalpel, and then a flexible freer was used to detach the fibrous tissue from the median nerve and the TCL; and (5) the TCL cutting blade was pushed straight to cut the TCL completely from distal to proximal. TCL length was observed until the complete release. The median nerve and the recurrent branch of the median nerve were observed.

**Results:**

All TCL were cut completely. All median nerves, recurrent branches of the median nerve, and superficial palmar arches could be observed during the operation, and none were injured. This technique showed effectiveness and safety for minimally invasive carpal tunnel surgery.

**Conclusions:**

The study found that the new device, MIS-CTS kits, along with this technique is effective for CTS release in terms of minimally invasive open carpal tunnel surgery.

## 1. Introduction

Carpal tunnel syndrome is common and has a high prevalence of 6–10% in the elderly population with an average age of 54 years. It is twice more common in women than in men [1–6]. The initial treatment of mild symptoms can be conservative. However, if symptoms are more frequent or persistent, including muscular weakness and decreased handgrip or drooping, surgical treatment may be required; 20% of people with the disease need surgery [7–11]. Standard open surgery is a basic procedure with a 3–5 cm long incision and produces successful outcomes of carpal tunnel release, but wound complications are more frequent than with minimally invasive carpal tunnel surgery. The most common wound complications are wound infection, inflammation, wound dehiscence, and painful scar [9, 12–15]. Minimally invasive carpal tunnel surgery techniques, such as endoscopic surgery, have less wound complications and patients can return to work faster than with the standard open technique; however, minimally invasive carpal tunnel surgery has other complications, including recurrent incidents and incomplete release [12, 16–19]. Minimally invasive carpal tunnel surgery was developed to improve visualization. It is a surgical tool that is important for complete release [20–23].

**Figure 1:**
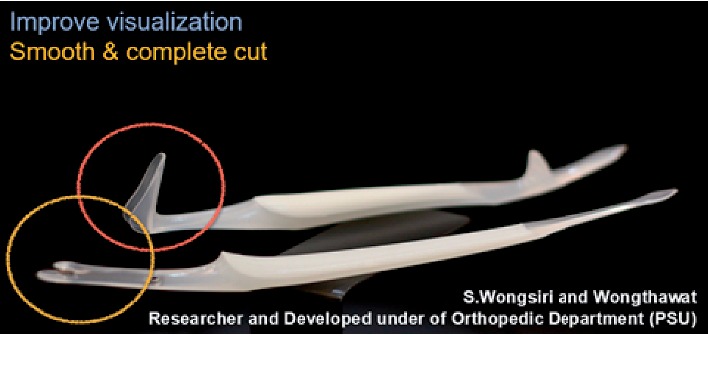
The minimally invasive surgery for carpal tunnel syndrome kits (MIS-CTS kits).

Nowadays, the medical costs are high, because of expensive surgical devices; the cost of endoscopic surgery carpal tunnel surgery is higher than that of standard open and other minimally invasive methods [24, 25]. The complexity of tools and the surgical technique of endoscopic surgery require a longer learning curve for surgeons [26–28]. In order to eliminate the difficulties of use and cost, the first generation (PSU-CTR®) and the second generation (named MiniSURE®) of MIS-CTS kits were developed to help patients gain minimally invasive surgery outcomes (Figure 1). Now, surgery is easier because surgeons have greater visibility [22, 23, 29]. In previous studies of both generations of MIS-CTS kits, we found better outcomes with small wounds, less pain, and early return to work [29]. Nonetheless, we have improved some features in the next generation of kits, thus increasing effectiveness and safety. The new features include improved hand griping, antislipping with expandable visual enhancer, and insertion length of marker of TCL cutting blade and flexible freer. The purposes of this research are to study the effectiveness of visualization during surgery and the complete release of TCL and also the safety of using the MIS-CTS kits.

## 2. Methods

### 2.1. Study Subjects

**Figure 2:**
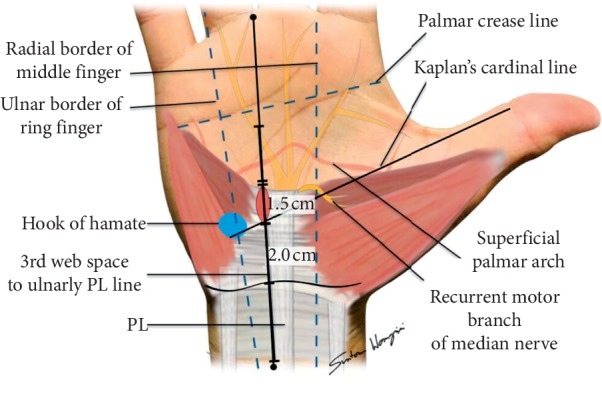
The landmark of anatomy of hand using Kaplan's cardinal line.

This study was approved by the Institutional Review Board of Faculty of Medicine, Prince of Songkla University (IRB number EC 60-282-11-1). This study was performed on 20 forearms from 10 fresh cadavers obtained within 72 hours after death. The cadavers came from a donation center of faculty of science, Prince of Songkla University. The operation was performed by the same author. Procedures were performed in the supine position and described the landmark by Kaplan's cardinal line (Figure 2).

### 2.2. The Surgical Technique

**Figure 3:**
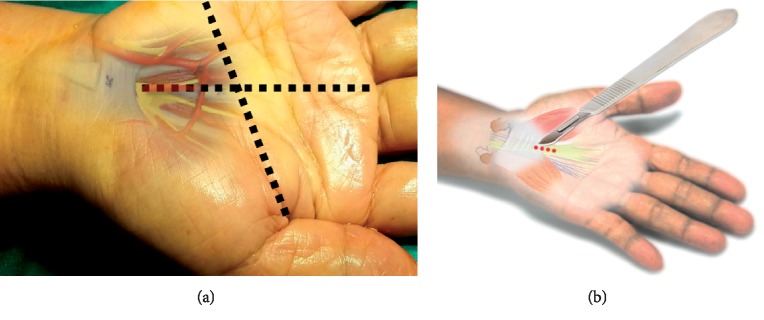
The first step for the author's surgical technique using MIS-CTS kits.

The incision direction is the line between the middle finger and ring finger along the wrist crease. Incision of 15–18 mm, starts away from the wrist crease about 2 to 2.5 cm distally. (Figure 3)

**Figure 4:**
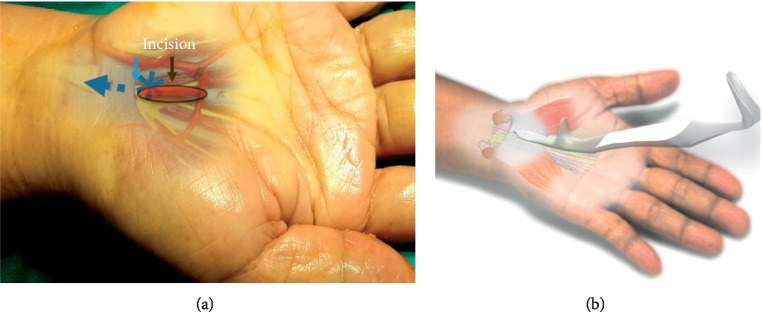
The second step for the author's surgical technique using MIS-CTS kits.

The Palmaris longus tendon is retracted by Senn retractors, and then the TCL is observed at the base. Scissors are used to create working space underneath the PL tendon and palmar aponeurosis. Then, insert the navigator to increase the size of the space for visual enhancer insertion (Figure 4).

**Figure 5:**
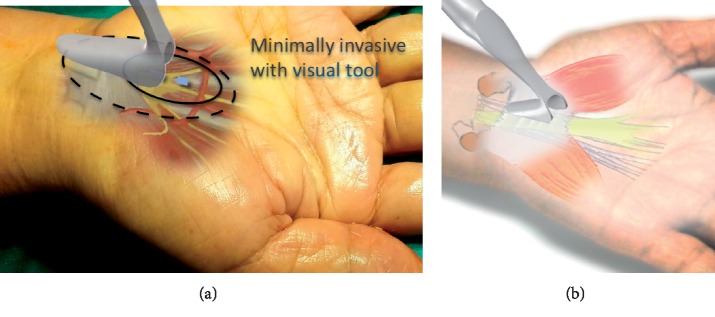
The third step for the author's surgical technique using MIS-CTS kits.

The visual enhancer is inserted in the space beneath the PL tendon and palmar aponeurosis. The transverse fiber of the TCL is observed from the bottom view. The visual enhancer improves the visual field by shielding the soft tissue around the operative field (Figure 5).

**Figure 6:**
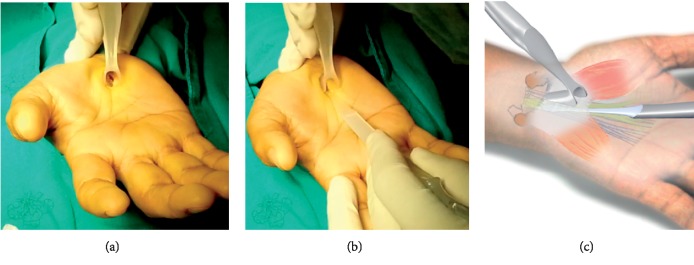
The fourth step for the author's surgical technique using MIS-CTS kits.

The TCL is cut by using a scalpel longitudinally at the top to create the long groove for the cutting guide and reducing the over prominence of TCL. The TCL is cut at the distal part for opening entry; then, a flexible freer is used to detach the fibrous tissue from the median nerve and the TCL (Figure 6).

**Figure 7:**
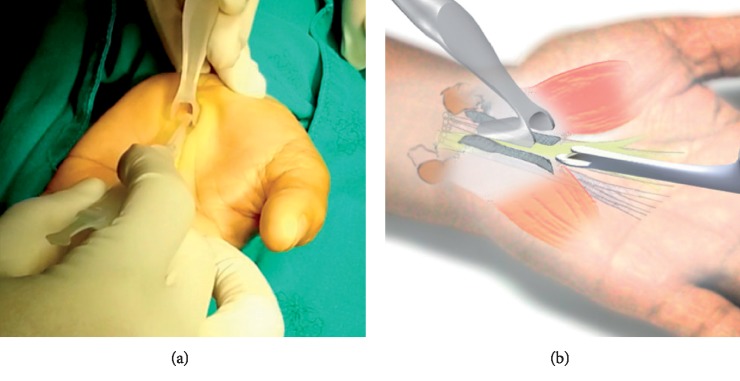
The fifth step for the author's surgical technique using MIS-CTS kits.

The TCL cutting blade is pushed straight to cut the TCL from the distal to proximal along the groove until the antebrachial fascia that is 2 to 2.5 centimeters proximal to the wrist crease. (Figure 7). Finally, complete release can be directly checked by direct vision via the visual enhancer or through the use of a probe with the MIS-CTS kits, the TCL can be seen clearly through the small incision.

### 2.3. Statistical Analysis

The descriptive statistics was used in this study. Cadaveric data collected included sex, age, side of hand, transverse carpal ligament length, incision site for single portal, type of release (complete release/incomplete release), median nerve injury (yes/no), type of recurrent branch of median nerve, and recurrent branch of median nerve injury.

## 3. Results

This study was from 20 forearms from 10 fresh cadavers: age of 20 forearms ranged from 58–82 years (average 73.8 years); fresh cadavers were 4 male and 6 female; and forearms were 10 right and 10 left. The incision for Mini-CTS was 15–18 mm (average 16.2 mm). Transverse carpal ligaments were 26–36 mm (average 31 mm). All Mini-CTS of carpal tunnel release were complete with no median nerve injury and no recurrent branch of median nerve injury (Table 1). The recurrent branches of the median nerve, 13 were type A (65% in the extraligamentous type), 6 were type B (30% in the subligamentous branching type), and 1 were type C (5% in the transligamentous type) (Table 2). This technique was complete release TCL in all forearms.

## 4. Discussion

The new minimally invasive carpal tunnel release technique, the author's technique, using MIS-CTS kits, and enhanced visualization, offered a promising result in all 20 cadavers with complete release of TCL and no nerve or vascular injury. The modified minimally invasive technique using visual enhancer could improve the visualization for surgery. This technique can avoid incisions on the touching zone of the hand, 2–2.5 cm from the wrist crease, which is a sensitive area for touching and bending. The touching zones are composed of the palmar cutaneous branches of the median nerve, which lie superficial to the transverse carpal ligament [28, 30–33]. Palmar area operative scar may be related to the cause of chronic persistent pain, pillar pain, and slow return to work [14, 34–37]. Currently, the minimally invasive technique of carpal tunnel release is popular for patients due to its benefits and satisfaction.

In the previous study, the minimal invasive surgery of 1.5–1.8 cm skin incision was performed with the visual enhancer [22]. It showed significantly better maximal visual length compared with the Senn retractor (47.7 (8.1) mm vs. 39.2 (6.5) mm). For careful attention of safety, the author's approach by the distal approach can gain benefit to observe directly in the superficial palmar vascular arch and recurrence branch of the median nerve. Also, when the TCL cutting blade is pushed straight from the distal to the proximal end of TCL, it can cut the TCL all at once.

In contrast, the endoscopic technique needs to cut TCL many times because the scalpel is designed to cut by layers from the bottom to the top. Sometimes, surgeons may have difficultly determining the other fascia from TCL when looking from the bottom of the endoscope view [30, 38]. The brachial fascia look like TCL, and it also continues from TCL.

The author observed that the TCL cutting blade offered the promising result of complete detachment, and the smooth cutting edge did not fray any remnants. The author believes that the smooth cutting edge, without fraying remnants of TCL and both edges, can reduce the risk of recurrence. Usually, TCL requires many attempted cuts, such as using the scissors or the endoscope, which may risk fraying remnants with the rough edge of TCL. The author's technique using a scalpel is more effective. However, more research is required in order to determine the risks of recurrence, thus improving patient outcome. Because the lower tip is slim and short, pressure in the carpal tunnel is reduced. The concept of less pressure with a slim and short tip may be the right way to reduce nerve injury while inserting equipment; this aspect needs to be studied further. Most advice states not to insert an endoscope if there is severe compression or the tunnel is really tight. Because of a long incision of standard open CTS release, the complications and sequelae are mainly wound problems, scar pain, wound dehiscence, and slow return to work [9, 13, 31, 32]. The minimally invasive technique was introduced for improving wound complication.

Minimally endoscopic carpal tunnel release is used to minimize wound problems. The endoscopic technique can approach from either portal 2 or portal 1 and makes an impressive minimal skin incision of 1.5 cm [16, 18, 33, 39]. The meta-analysis of randomized controlled trials in the endoscopic versus open carpal tunnel release for idiopathic carpal tunnel syndrome significantly reduced postoperative hand pain, it increased the possibility of reversible postoperative nerve injury in patients with idiopathic CTS but statistical difference in the overall complication rate, subjective satisfaction, the time to return to work, postoperative grip and pinch strength, and operative time. However, the endoscope is an imaging and IT technology tool that needs special training with a longer learning curve [40]. Many studies found that transient nerve problems may occur during operations, especially inserting dilators or metallic equipment in the pressured carpal tunnel. The endoscope is not a proper device to use in severe compression. Once the surgeon finds the obstacle during insertion of the tool, the surgeon must stop and convert to open carpal tunnel surgery because the median nerve may be damaged or torn.

The Indiana tome is a minimally invasive carpal tunnel surgery that uses local anesthesia and approaches the median nerve from the palm using a special scalpel to cut the TCL [41, 42]. The Indiana tome simplified carpal tunnel surgery. It transformed carpal tunnel surgery to a faster, same day procedure. It seems the Indiana tome and other carpal tunnel tools, such as the Safeguard and Knight light, offer promising results, including complete TCL dividing, short incision, short operative time, and early return to work. However, the literature review highlights some nerve problems that can occur while using this equipment, even though some new developments have reduced the size of equipment [41, 43, 44]. We have developed both a new equipment and a new technique to avoid using the cannula and dilator, which may increase pressure on the carpal tunnel. We believe that minimal insertion in high-pressured carpal tunnel will reduce the risk of transient neuropathy; however, further clinical studies are required. The AAOS published recommendations for CTS: visualization and completely dividing TCL are the major roles of CTS [45]. Authors also believe the same important points. Currently, we are developing a visualization tube for improving visualization and a specific TCL knife for complete release. It is possible to improve the technique and the tool. This technique and the tool can perform the minimally invasive technique [22, 23]. It offers a simplified technique with good outcomes of improved visualization and complete release. For the benefit of patients, it should reduce operative pain, touching scar pain, wound complication, and transient neuropathy and offer an early return to work. For the benefit of surgeons, the distal incision made using the special visual enhancer should improve visualization and improve safety while preventing injury of the median nerve, the ulnar-median nerve palmar communicating branch, and the superficial palmar arch. For the benefit of the healthcare system: it may reduce the currently critically high costs of surgery. In 2002, Martin reported that CTS cost 4 billion dollars annually. In 2003, the increasing cost of healthcare is 12%. The healthcare system could save from lower costs for equipment, minimal equipment usage, shortened operative service costs, no anesthetic team, one surgeon, and one nurse.

The author's technique and the novel tool can improve effectiveness from the original standard of open release of CTS with both practical benefits (simple to use, enhanced visualization, complete cut) and productivity benefits (MIS, save time, save cost, and quick return to work). In the current era of high-cost healthcare, the author hopes that author's method will provide affordability for more patients to receive quality service. If any surgical technique and equipment is developed with the same concept of value, the healthcare costs will be reduced for patients, including health insurance and government support. This is the only cadaveric study which proves the concept. However, the author believes that a clinical study is important for the next stage.

## 5. Conclusion

This study found that the author's device (MIS-CTS kits) with the author's technique was effective and safe for TCL completely release in terms of minimally invasive open carpal tunnel surgery.

## Figures and Tables

**Table 1:** The results of carpal tunnel released by the MIS-CTS kits.

Sex	Cadaveric forearm (N)	Average age (years)	Average incision (mm)	Type of release (complete/incomplete)	Average TCL length (mm)	Median nerve injury (%)	Recurrent branch of median nerve injury (%)
Male	Left (4) Right (4)	67	16	Complete (100%)	32.5	0	0

Female	Left (6) Right (6)	78	16.63	Complete (100%)	30	0	0

Total	20	73	16.2	Complete (100%)	31	0	0

**Table 2:** Type of recurrent branches of the median nerve.

Type	*N* (%)
Type A (extraligamentous type)	13 (65)
Type B (subligamentous branching type)	6 (30)
Type C (transligamentous type)	1 (5)

## Data Availability

No external data are applicable to this manuscript.

## Abbreviations

TCL: Transverse carpal ligament
CTS: Carpal tunnel syndrome
OCTR: Open carpal tunnel release
ECTR: Endoscopic carpal tunnel release
MIS-CTS kits: Minimally invasive surgery for carpal tunnel syndrome kits.
